# Supplementary material for: Randomized Phase 2 Clinical Trial of Olaratumab in Combination with Gemcitabine and Docetaxel in Advanced Soft Tissue Sarcomas
Source: Cancers (Basel). 2023 Oct 6;15(19):4871. doi: 10.3390/cancers15194871 (PMC10572019; doi:10.3390/cancers15194871)
Supplement: Supplementary file 1 [file cancers-15-04871-s001.zip › cancers-2557282-supplementary.pdf]

Supplementary Materials

Supplementary Table S1. Overall survival by histological tumor type (LMS and non-LMS).

		Olaratumab-naïve			Olaratumab-pretreated		
Arm		Investigational arm (O+G+D) N=81	Control arm (PBO+G+D) N=86		Investigational arm (O+G+D) N=46	Control arm (PBO+G+D) N=43	
Histological tumor type		Median[a] (95% CI)		HR[b] (95% CI) [*P*-value[c]]	Median[a] (95% CI)		HR[b] (95% CI) [*P*-value[c]]
Median OS	LMS	17.3 months (15.3–34.8)	22.0 months (12.9–28.5)	0.82 (0.46–1.45) [0.48]	31.7 months (17.9–-)	20.3 months (15.8–27.1)	0.50 (0.20–1.23) [0.12]
	Non-LMS	16.2 months (12.2–24.1)	15.1 months (10.6–21.6)	0.96 (0.58–1.59) [0.86]	18.8 months (6.6–-)	11.2 months (6.8–20.2)	0.75 (0.37, 1.50) [0.41]

Abbreviations: CI = confidence interval; D = Docetaxel; G = Gemcitabine; HR = Hazard ratio; N = number of patients in population; n = number of patients in the specified category; O = Olaratumab; OS = Overall Survival. [a] Estimated using the Kaplan-Meier method. [b] Hazard ratio (Treatment A vs Treatment B) and 95% CI (Wald) were estimated from unstratified Cox model. [c] Two-sided p-value from unstratified log-rank test

Supplementary Table S2. Overall survival and progression-free survival by histological tumor type (liposarcoma, leiomyosarcoma, undifferentiated pleomorphic sarcoma and other).

	**Overall Survival[a]**			**Progression-free Survival[a]**		
**Histological tumor type**	**Investigational arm (O+G+D)**	**Control arm (PBO+G+D)**		**Investigational arm (O+G+D)**	**Control arm (PBO+G+D)**	
	**Median[b]**		**HR[c] (95% CI) [*P*-value[c]]**	**Median[b]**		**HR[c] (95% CI) [*P*-value[d]]**
**Liposarcoma**	N=16<br>14.6 months	N=14<br>NE	1.63 (0.59–4.48)<br>[0.324]	N=16<br>8.1 months	N=14<br>4.4 months	1.04 (0.44–2.47)<br>[0.923]
**Leiomyosarcoma**	N=58<br>27.0 months	N=58<br>21.1 months	0.73 (0.45–1.17)<br>[0.188]	N=58<br>8.2 months	N=58<br>5.8 months	0.70 (0.44–1.12)<br>[0.137]
**Undifferentiated Pleomorphic Sarcoma**	N=10<br>20.4 months	N=13<br>15.9 months	0.56 (0.19–1.64)<br>[0.284]	N=10<br>5.5 months	N=13<br>5.9 months	0.64 (0.20–2.06)<br>[0.452]
**Other**	N=43<br>16.2 months	N=44<br>11.1 months	0.83 (0.50–1.37)<br>[0.455]	N=43<br>4.4 months	N=44<br>4.0 months	0.73 (0.46–1.17)<br>[0.192]

Abbreviations: CI = confidence interval; D = Docetaxel; G = Gemcitabine; HR = Hazard ratio; N = number of patients in population; NE = not estimable; O = Olaratumab; OS = Overall Survival. [a] O-naïve and O-pretreated cohorts were pooled for this analysis. [b] Estimated using the Kaplan-Meier method. [c] Hazard ratio (Investigational vs Control arm) and 95% CI (Wald) were estimated from unstratified Cox model. [d] Two-sided p-value from unstratified log-rank test

Supplementary Table S3. Overall survival and progression-free survival by treatment and PDGFR status (positive/negative) in the total soft tissue sarcoma population.

			Overall Survival			Progression-free Survival		
**Arm**			**Investigational arm (O+G+D)**	**Control arm (PBO+G+D)**		**Investigational arm (O+G+D)**	**Control arm (PBO+G+D)**	
	**PDGFR status[a]**		**Median[b]**		**HR[c] (95% CI) [*P*-value[d]]**	**Median[b]**		**HR[c] (95% CI) [*P*-value[d]]**
**Olaratumab-naïve**	**PDGFR-α N=155**	**(–) n=62**	16.8 months	19.7 months	0.96 (0.5–1.84) [0.905]	8.2 months	6.9 months	0.71 (0.39–1.3) [0.273]
		**(+) n=93**	16.2 months	15.8 months	0.97 (0.6–1.59) [0.916]	7.0 months	4.0 months	0.79 (0.49–1.27) [0.331]
	**PDGFR-β N=154**	**(–) n=45**	16.8 months	19.9 months	0.68 (0.33–1.41) [0.301]	6.9 months	4.2 months	0.85 (0.43–1.68) [0.649]
		**(+) n=109**	16.2 months	18.0 months	1.07 (0.67–1.71) [0.781]	7.6 months	4.4 months	0.72 (0.46–1.13) [0.157]
**Olaratumab-pretreated**	**PDGFR-α N=74**	**(–) n=29**	19.6 months	20.2 months	0.87 (0.34–2.21) [0.766]	5.2 months	8.0 months	1.65 (0.6–4.53) [0.332]
		**(+) n=45**	19.3 months	13.1 months	0.60 (0.28–1.28) [0.185]	5.5 months	2.8 months	0.72 (0.37–1.4) [0.333]
	**PDGFR-β N=73**	**(–) n=20**	19.6 months	19.2 months	0.88 (0.29–2.65) [0.824]	5.2 months	4.1 months	1.04 (0.35–3.11) [0.938]
		**(+) n=58**	19.8 months	19.1 months	0.72 (0.36–1.44) [0.348]	5.5 months	5.4 months	0.91 (0.48–1.72) [0.767]

Abbreviations: + = positive; – = negative; CI = confidence interval; D = Docetaxel; G = Gemcitabine; HR = Hazard ratio; N = number of patients in population; n = number of patients in the specified category; O = Olaratumab; OS = Overall Survival. [a] PDGFR-α tumor status was determined with a rabbit monoclonal antibody (Cell Signaling Technology clone D13C6) specific for PDGFR-α without cross-reactivity to PDGFR-β. PDGFR-β tumor status was determined with a mouse monoclonal antibody (Cell Signaling Technology clone 2B3) proven to be specific for PDGFR-β with no

cross-reactivity for PDGFR-α. The status for PDGFR-α and PDGFR-β was provided as a dichotomous variable with “positive” and “negative” expression, where a “positive” result showed ≥10% of the tumor as demonstrating at least weak but specific membranous expression (1+ on a 0, 1+, 2+, 3+ scale of staining intensity). “Negative” corresponded to expression that did not meet these criteria. [b] Estimated using the Kaplan-Meier method. [c] Hazard ratio (Investigational vs Control arm) and 95% CI (Wald) were estimated from unstratified Cox model. [d] Two-sided *P*-value from unstratifed log-rank test

Supplementary Table S4. Summary of patient-reported outcomes (QLQ-C30 global health status/QoL subscale and mBPI-sf scores) for the O-naïve and O-pretreated cohorts.

	Olaratumab-naïve			Olaratumab-pretreated		
**Arm**	**Investigational arm (O+G+D) N=81**	**Control arm (PBO+G+D) N=86**		**Investigational arm (O+G+D) N=46**	**Control arm (PBO+G+D) N=43**	
**Patient-reported outcomes**			HR (95% CI) [*P*-value*]			HR (95% CI) [*P*-value*]
**QLQ-C30 global health status/QoL subscale**	**n=81**	**n=86**		**n=45**	**n=43**	
Mean baseline scores (SD)	68.5 (19.7)	68.7 (23.1)		72.6 (19.7)	69.8 (21.4)	
Median time to worsening, months (95% CI)	1.1 (0.8–1.5)	1.8 (0.8–2.8)	1.21 (0.82–1.79) [0.32]	1 (0.8–3.9)	1.5 (0.8–2.9)	1.11 (0.63–1.95) [0.79]
**mBPI-sf**	**n=67**	**n=71**		**n=41**	**n=34**	
Mean baseline scores for worst pain (SD)	3.0 (2.6)	2.5 (2.8)		2.4 (2.7)	1.8 (2.5)	
Median time to first worsening, months (95% CI)	3.6 (2.7–8.6)	2.3 (1.4–6.5)	[0.07]	3.2 (1.4–7.6)	2.2 (0.76–3.02)	[0.23]

Abbreviations: CI = confidence interval; D = Docetaxel; G = Gemcitabine; HR = Hazard ratio; mBPI-sf = modified Brief Pain Inventory-short form; N = number of patients in population; n = number of patients in the specified category; QLQ-C30 = the European Organization for Research and Treatment of Cancer (EORTC) quality of life questionnaire; QoL = quality of life; O = Olaratumab; OS = Overall Survival; SD = standard deviation. **P*-value was analyzed per stratified log-rank test.

CONSORT 2010 Flow Diagram

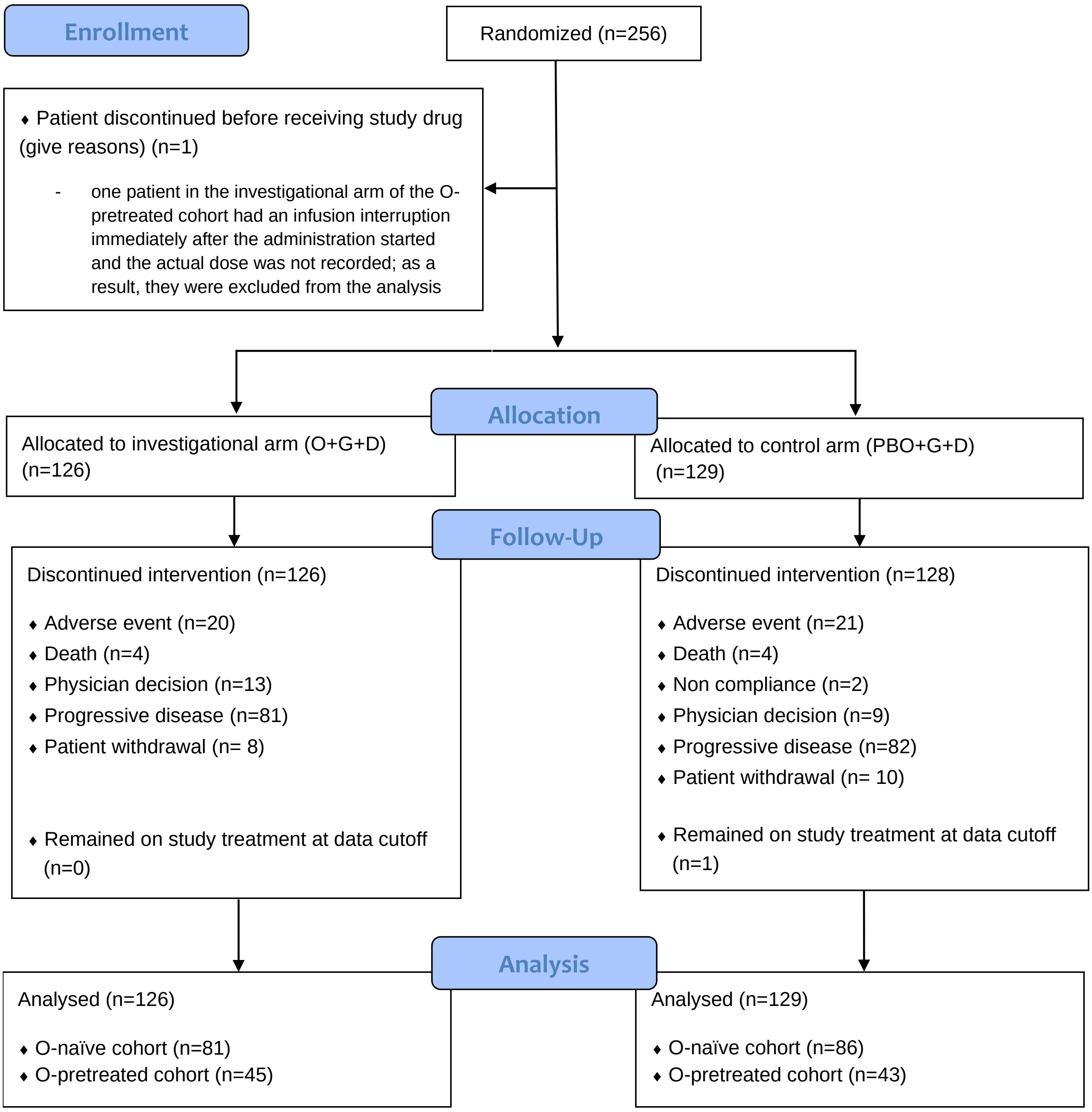

Supplementary Figure S1. CONSORT Flow Diagram of the ANNOUNCE 2 Trial.

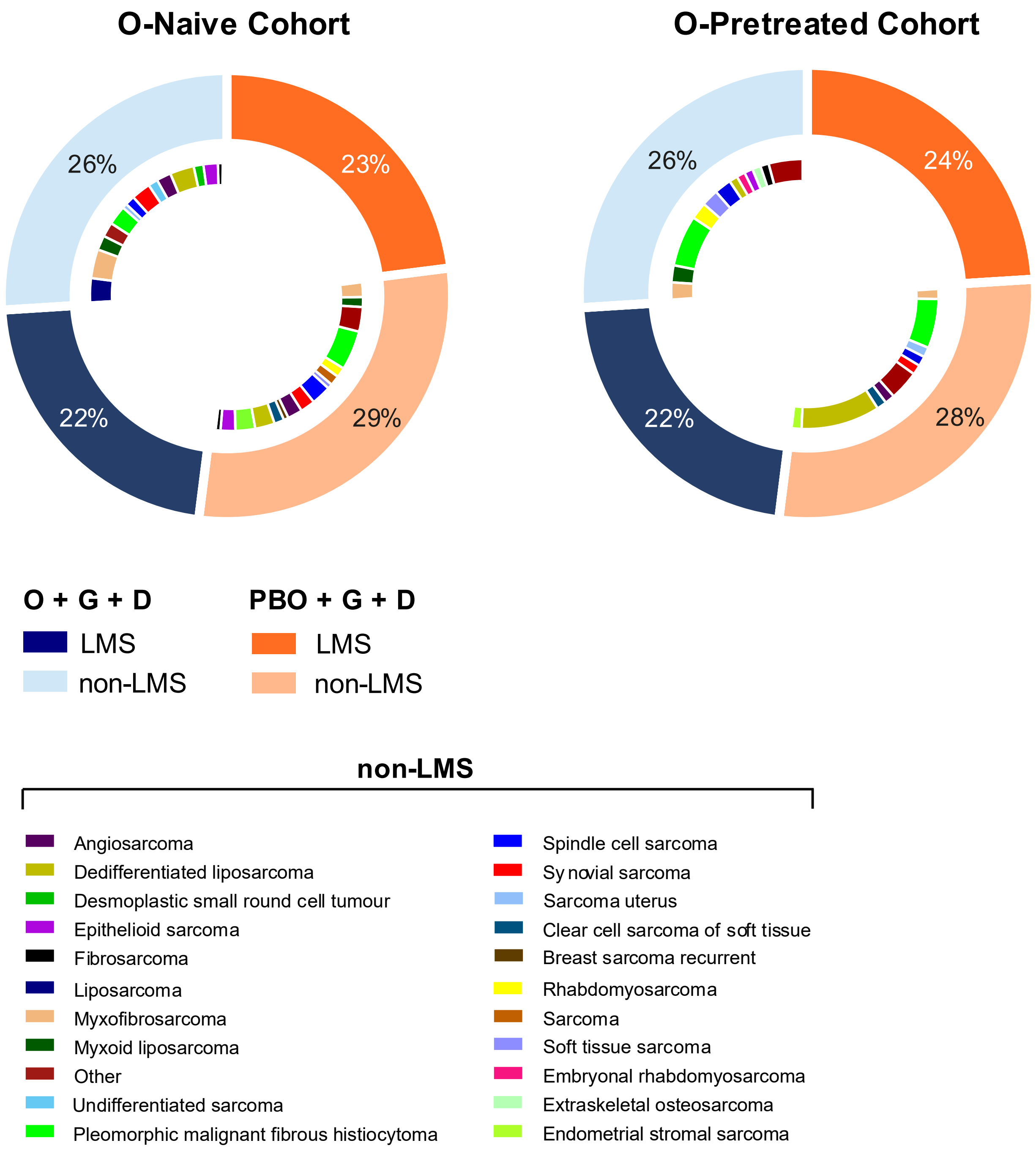

Supplementary Figure S2. Soft Tissue Sarcoma Subtypes in the O-naïve and O-pretreated cohorts.

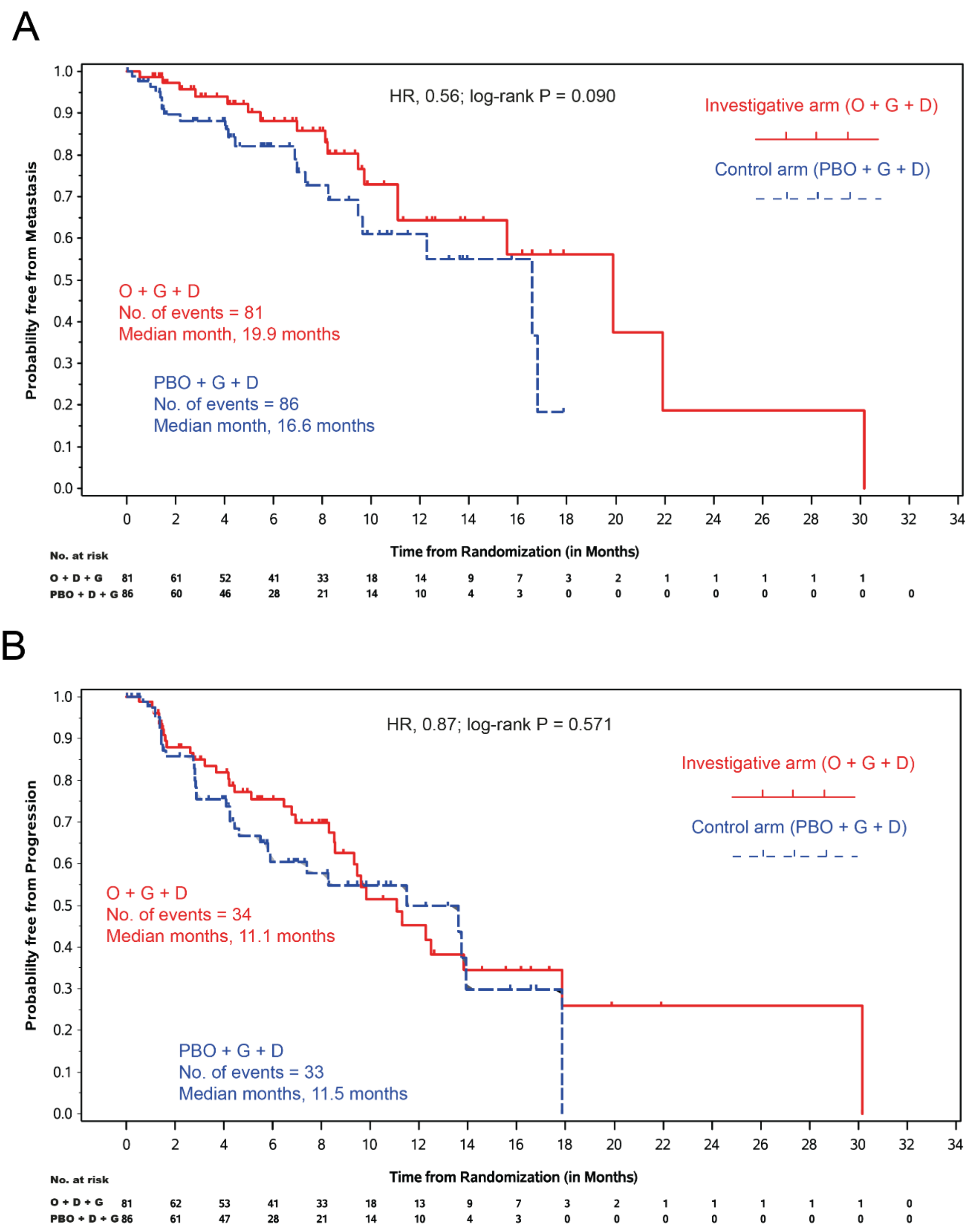

Supplementary Figure S3. KM Plots of time to (A) any metastasis and (B) any progression based solely on increased sum of target lesions, for the O-naïve cohort (ITT population).

Abbreviations: D = Docetaxel; G = Gemcitabine; HR = Hazard ratio; ITT = intent to treat population; KM = Kaplan-Meier; No. = number; O = Olaratumab; PBO = placebo
